# Effectiveness of incisional negative pressure wound therapy after major lower extremity amputation: a randomised controlled trial

**DOI:** 10.1308/rcsann.2023.0011

**Published:** 2023-07-12

**Authors:** VV Vaddavalli, B Girdhani, A Savlania, A Behera, A Rastogi, L Kaman, K Abuji

**Affiliations:** Post Graduate Institute of Medical Education and Research, Chandigarh, India

**Keywords:** Amputation, Surgical site infection, PAD, iNPWT, Stump dehiscence, RCT

## Abstract

**Introduction:**

The aim was to study the effect of incisional negative pressure wound therapy (iNPWT) in wound healing compared with standard sterile gauze dressings after major lower extremity amputation in patients with peripheral arterial disease (PAD).

**Methods:**

This prospective, randomised controlled trial included 50 patients undergoing major lower extremity amputations for PAD. Patients were randomised into iNPWT and standard dressing groups. The patency of blood vessels at the level of the stump was ensured with or without revascularisation. The primary outcome was wound-related complications such as surgical site infection (SSI), wound dehiscence, seroma/haematoma formation or the need for revision amputation. The secondary outcome was the time taken for the eligibility of prosthesis placement.

**Results:**

It was found that only 12% of the patients in the iNPWT group had SSI compared with 36% in the standard dressing group (*p* = 0.047). Rates of wound dehiscence, seroma/haematoma formation and revision amputation were decreased in the iNPWT group but this was not statistically significant (*p* > 0.05). There was a significant reduction in the time taken for eligibility of prosthesis placement in the iNPWT group (5.12 ± 1.53 vs 6.8 ± 1.95 weeks, *p* = 0.002).

**Conclusions:**

iNPWT is effective in reducing the incidence of SSI and the time taken for rehabilitation in patients undergoing major lower limb amputation due to PAD.

## Introduction

Amputation is an age-old surgery first described by Hippocrates.^1^ Approximately 185,000 people undergo lower extremity amputation in the USA annually.^2^ Peripheral arterial disease (PAD) is the most common cause of major limb amputation worldwide.^3^ The complications of amputation may include surgical site infection (SSI), stump haematoma/seroma formation, stump dehiscence, stump necrosis and the need for revision amputation. SSIs occur in up to 40% of patients following major lower extremity amputations.^4^ SSIs, haematoma and seroma formation increase the risk of stump dehiscence and the need for revision amputation. This leads to increased patient morbidity.^5^

In patients with PAD, many risk factors cause delayed wound healing, such as a history of smoking, diabetes mellitus and decreased perfusion to the wound. In an earlier study, approximately 10%–20% of patients who undergo a below knee amputation (BKA) eventually need an above knee amputation (AKA), particularly patients with diabetes mellitus (DM) or PAD.^6^

Negative pressure wound therapy (NPWT), popularly known as vacuum-assisted closure, has been used to treat acute and chronic wounds for two decades having been introduced by Argenta and Morykwas in the 1990s. NPWT refers to the application of sub-atmospheric pressure over the wound, either intermittently or continuously.^7,8^ NPWT has shown promising results in wound healing in patients with PAD.^9^ The use of NPWT over closed incisions is known as incisional negative pressure wound therapy (iNPWT). In their meta-analysis, Semsarzadeh *et al* studied the benefits of iNPWT over abdominal, chest, groin and lower extremity trauma incisions and found a 29.4% relative reduction in SSI.^10^ The benefits of iNPWT after major lower extremity amputation for PAD have not yet been established in a prospective study. The reason for the current study was to look for the efficacy of iNPWT on wound healing after major lower extremity amputation when compared with standard sterile gauze dressings.

## Methods

### Study design

This prospective randomised controlled trial (RCT) was conducted after registration with Clinical Trials Registry – India (CTRI/2021/02/031166). Written informed consent was obtained from all participants, and the study was conducted in accordance with the Declaration of Helsinki. Ethical clearance was obtained from the institutional ethical committee (NK/6602/MS/733).

### Participants

Fifty patients who underwent major lower extremity amputation for PAD because of non-salvageable limbs were included. This study was conducted between February and August 2021 at the Post Graduate Institute of Medical Education and Research, a government-funded tertiary care centre in northern India. Patients with PAD who underwent major lower extremity amputation with at least one patent artery for adequate blood supply to the stump, with or without revascularisation, were included. In the case of AKA, patency of the profunda femoris artery, and in BKA, patency of at least one tibial artery was ensured using computed tomography angiography (preoperative) or arterial duplex ultrasonography (pre- and postoperative). In patients requiring revascularisation, inflow correction was ensured by surgical bypass (aorto-femoral or femoro-popliteal/tibial or femoro-femoral crossover bypass) or endovascular revascularisation (iliac artery stenting, femoro-popliteal or tibial artery angioplasty). Patients with inadequate lower extremity perfusion, glycated haemoglobin (HbA_1c_) > 12%, who underwent lower extremity amputation for collagen vascular disease, malignant ulcer and untreated osteomyelitis were not included. Patients who underwent guillotine amputation and did not give consent were also not included.

### Intervention

The surgical site was prepared with chlorhexidine solution before the incision (3M Skin Prepping antiseptic solution chlorhexidine gluconate I.P. 2% w/v and isopropyl alcohol I.P. 70% v/v; 3M India Limited, Pune, India). A prophylactic antibiotic, intravenous cefuroxime or if allergic to penicillin, vancomycin was given to all patients 30min before skin incision. If the patient was in sepsis preoperatively, broad-spectrum antibiotics were given according to the therapeutic schedule. After fascial closure, the subcutaneous layer was approximated using polydioxanone 2-0 interrupted sutures (PDS II; Ethicon Inc., Mexico). For skin closure, nylon 2-0 interrupted, vertical mattress sutures (Ethilon; Ethicon, Johnson & Johnson Pvt. Ltd, Aurangabad, India) were used. Antibiotic-coated sutures were not used. We did not use a drain as per institutional protocol. All amputations were performed under the direct supervision of a single attending vascular surgeon.

After skin closure, the incision was cleansed and dried with a sterile gauze. For patients in the iNPWT group, a closed incision NPWT system (CCNPWT^®^; Triage Meditech Pvt Ltd, New Delhi, India) was applied as per the manufacturer’s instructions. The incision was covered with a silicone wound contact layer on which a polyurethane foam sheet was applied. Over this a transparent, adhesive polyurethane film was applied, ensuring an adequate seal. It was then connected to a suction pump, and continuous negative pressure of 125mmHg was applied. The iNPWT was removed on the sixth postoperative day. For patients in the standard dressing group, a sterile non-adherent petroleum-based dressing along with sufficient padding wrapped with sterile gauze was applied, and removed postoperatively after 48h. Depending on the condition of the stump, further dressings were applied. For patients who underwent BKA, the knee was maintained in a functional position using a knee immobiliser to prevent contracture, and intermittent active and passive flexion and extension exercises at the knee joint were performed.

### Outcomes

The primary outcome of this study was wound-related complications such as SSI, wound dehiscence, seroma/haematoma formation and the need for revision amputation. The secondary outcome was the time taken for the eligibility of prosthetic placement. We assessed SSIs clinically according to the criteria of the Centers for Disease Control and Prevention.^11^ If any wound complication occurred, it was treated according to the hospital’s protocol. Device-related complications such as skin blistering, negative pressure-related pain and superficial skin necrosis were also assessed. The outcomes were evaluated by two physicians until the sixth day after surgery. After the sixth postoperative day, the wound was assessed by two wound-care specialists who were unaware of the randomisation to minimise potential bias. We followed the patients for a minimum of 8 weeks or until complete healing of the stump in cases of SSI. The baseline characteristics of study participants and their comorbidity profile were assessed at the time of admission and were recorded. Data were maintained prospectively in an electronic and paper-based case record form.

### Sample size

Calculation of the sample size was done based on the previously reported occurrence of wound infection. With an expected outcome of 6% in the intervention group compared with 40% in the control group, a power of 0.8, and a type I error of 0.05, we calculated a sample size of 25 per group with an anticipated loss of 10% to follow-up.

### Randomisation

**Figure 1:**
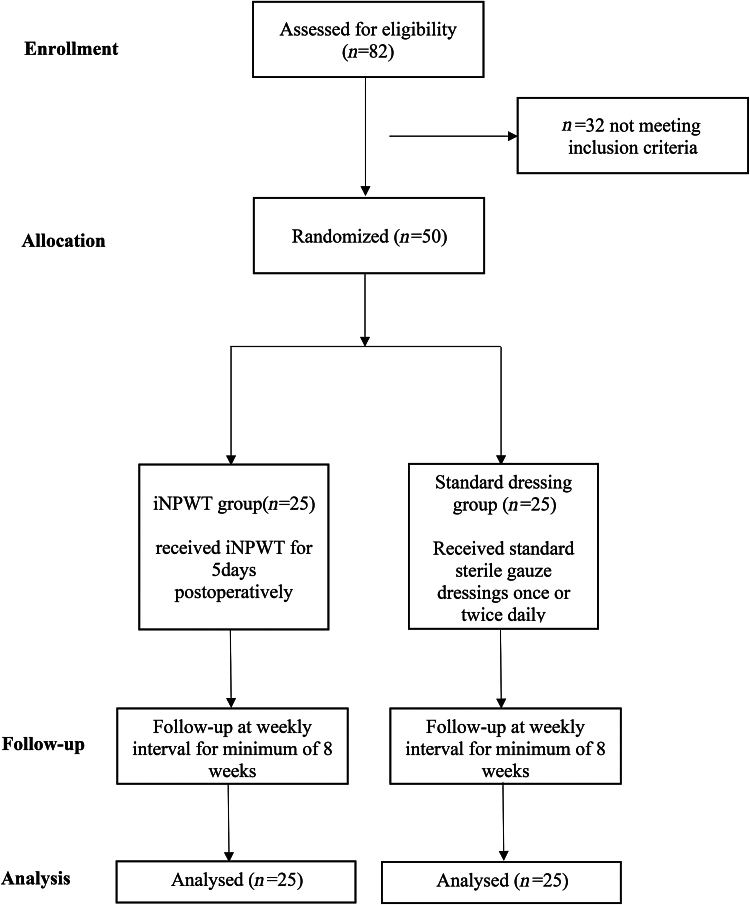
Flow chart showing the study design

Patients were randomised to iNPWT and standard dressing groups in a 1:1 ratio. The random allocation sequence was computer generated using block randomisation. Allocation concealment was done using sealed opaque envelopes. The study design is shown in Figure 1. Double blinding of the study was not possible because of the nature of the therapy. However, measures were taken to minimise potential bias in accordance with the recommendations of Karanicolas *et al* regarding blinding in surgical research.^12^

### Statistical analysis

Qualitative data were represented using numbers and percentages, and quantitative data were represented using mean ± standard deviation (sd) and median. The normality of the data was checked using the Shapiro–Wilk test. To make group comparisons, an unpaired *t*-test was used for quantitative data, and chi-squared and Fischer’s exact tests were used for qualitative data. For non-normally distributed data Wilcoxon–Mann–Whitney *U*-test was used to make group comparisons. A *p*-value < 0.05 was considered statistically significant. All statistical calculations were carried out using standard statistical programs (SPSS, Microsoft Excel).

## Results

The demographic details of the study population are shown in Table 1. The mean age was 55 ± 14 years; 74% were male, 52% were chronic smokers and 40% were alcohol dependent. Thirty-five patients (70%) had type 2 diabetes mellitus (T2DM), and 17 (34%) had hypertension. A history of coronary artery disease (CAD) and cerebrovascular accident (CVA) was present in 12% and 8% of patients, respectively. Five patients (10%) had end-stage renal disease (ESRD) and one patient had hypothyroidism. Among patients with T2DM, 24 of 35 (68.57%) had uncontrolled DM (HbA_1c_ > 7).

**Table 1:** Demographic details of patients in the study

Variables	*n* (%) (*N* = 50)
Age, years	55 ±14
Gender	
Male	37 (74.0)
Female	13 (26.0)
T2DM	35 (70.0)
HTN	17 (34.0)
CAD	6 (12.0)
CVA	4 (8.0)
ESRD	5 (10.0)
Hypothyroidism	1 (2.0)
Smoking	26 (52.0)
Alcohol	20 (40.0)

CAD = coronary artery disease; CVA = cerebrovascular disease; ESRD = end-stage renal disease; HTN = hypertension; T2DM = type 2 diabetes mellitus

HbA_1c_ (%) was not normally distributed. Mean and median values of HbA_1c_ were 7.34 and 7.00, respectively. Of 50 patients, 15 (30%) underwent AKA and 35 (70%) underwent BKA. Twelve patients had an SSI and nine had wound dehiscence. Three patients required revision amputation. The mean (±sd) time taken for eligibility of prosthesis placement was 6.18±2.18 weeks (Table 2).

**Table 2:** Outcomes in the study population

Outcomes	*n* (%) (*N* = 50)
Surgical site infection	12 (24.0)
Wound dehiscence	9 (18.0)
Seroma or haematoma formation	3 (6.0)
Revision amputation	3 (6.0)
Time taken for eligibility of prosthesis placement, weeks	6.18 ± 2.18

### Comparison between iNPWT and standard dressing groups

The comparison of demographic details, risk factors, comorbidities, and laboratory results between the two groups is shown in Table 3. There was no significant difference in the distribution of the above parameters between the two groups (*p* > 0.05). Twenty-four of 35 (68.57%) patients with T2DM had uncontrolled DM (HbA_1c_ > 7), 14 (58.3%) in the iNPWT group and 10 (41.6%) in the standard dressing group (*p* = 0.258). There was no significant difference between the two groups regarding distribution of patients with uncontrolled DM. Three of 25 (12%) patients had SSI in the iNPWT group compared with 9 of 25 (36%) in the standard dressing group (*p* = 0.047). There was a significant difference between the two groups regarding SSI.

**Table 3:** Comparison of parameters between two groups

Parameters	Group		*p*-value
	iNPWT (*n* = 25)	Standard dressing (*n* = 25)	
Age, years	57.60 ± 13.54	52.52 ± 13.60	0.192
Gender, *n* (%)			0.333
Male	20 (80.0)	17 (68.0)	
Female	5 (20.0)	8 (32.0)	
T2DM, *n* (%)	17 (68.0)	18 (72.0)	0.758
HTN, *n* (%)	6 (24.0)	11 (44.0)	0.136
CAD, *n* (%)	5 (20.0)	1 (4.0)	0.189
CVA, *n* (%)	2 (8.0)	2 (8.0)	1.000
ESRD, *n* (%)	1 (4.0)	4 (16.0)	0.349
Hypothyroidism, *n* (%)	1 (4.0)	0 (0.0)	1.000
Smoking, *n* (%)	14 (56.0)	12 (48.0)	0.571
Alcohol, *n* (%)	10 (40.0)	10 (40.0)	1.000
HbA_1c_, %	7.38 ± 1.49	7.30 ± 1.67	0.719
Haemoglobin, g/dl	9.76 ± 2.00	9.49 ± 2.51	0.678
TLC, mm^3^	15,904.00 ± 7,897.39	14,800.00 ± 7,903.27	0.535
Serum sodium, mEq/l	136.52 ± 3.84	136.64 ± 4.38	0.918
Serum potassium, mEq/l	4.35 ± 0.48	4.09 ± 0.57	0.096
Blood urea, mg/dl	37.36 ± 26.91	53.31 ± 43.04	0.207
Serum creatinine, mg/dl	1.08 ± 1.06	1.99 ± 1.93	0.116
Serum albumin, g/dl	2.79 ± 0.68	2.77 ± 0.63	0.884

CAD = coronary artery disease; CVA = cerebrovascular disease; ESRD = end-stage renal disease; HbA_1c_ = glycated haemoglobin; HTN = hypertension; iNPWT = incisional negative pressure wound therapy; T2DM = type 2 diabetes mellitus; TLC= total leukocyte count

**Figure 2:**
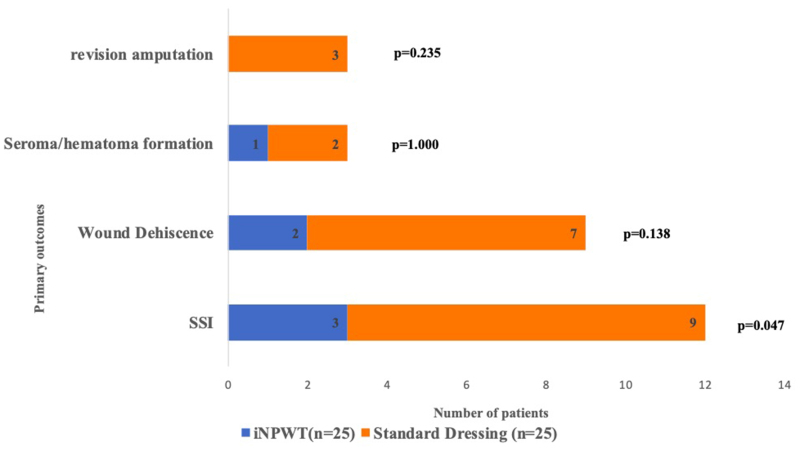
Wound-related complications between incisional negative pressure wound therapy and standard dressing groups

The parameters age, T2DM, hypertension (HTN), smoking and alcohol dependence were compared between patients who had SSI and those who did not. There was no significant difference between the two groups (*p* > 0.05) (Table 4). In patients with uncontrolled diabetes, 14 had an iNPWT dressing and only 1 had an SSI. The remaining ten patients underwent standard dressing; four of them developed an SSI (*p* = 0.0506). The percentage of wound dehiscence, seroma/haematoma formation and the need for revision amputation was lower in the iNPWT group but was not statistically significant (*p* > 0.05) (Table 5; Figure 2).

**Table 4:** Association of parameters with surgical site infection

	Surgical site infection		*p*-value
Parameters	Yes (*n* = 12)	No (*n* = 38)	
Age, years	53.42 ± 10.93	55.58 ± 14.52	0.776
T2DM, *n* (%)	7 (58.3)	28 (73.7)	0.471
HTN, *n* (%)	6 (50.0)	11 (28.9)	0.294
ESRD, *n* (%)	3 (25.0)	2 (5.3)	0.082
Hypothyroidism, *n* (%)	0 (0.0)	1 (2.6)	1.000
Smoking, *n* (%)	8 (66.7)	18 (47.4)	0.243
Alcohol, *n* (%)	4 (33.3)	16 (42.1)	0.740

ESRD = end-stage renal disease; HTN = hypertension; T2DM = type 2 diabetes mellitus

**Table 5:** Comparison of primary outcomes between two groups

Wound-related complications	Group		*p*-value
	iNPWT (*n* = 25)	Standard dressing (*n* = 25)	
Surgical site infection	3 (12.0)	9 (36.0)	**0.047** ^a^
Wound dehiscence	2 (8.0)	7 (28.0)	0.138^b^
Seroma or haematoma formation	1 (4.0)	2 (8.0)	1.000^b^
Revision amputation	0 (0.0)	3 (12.0)	0.235^b^

Values are given as *n* (%)

iNPWT = incisional negative pressure wound therapy

^a^Chi-squared test

^b^Fisher’s exact test

The time taken for complete healing of the stump and eligibility for prosthesis placement was 5.12 ± 1.53 weeks in the iNPWT group and 6.8 ± 1.95 weeks in the standard dressing group (*p* = 0.002). This shows a significant difference between the two groups in terms of time taken for rehabilitation. As mentioned in the Methods, complications related to iNPWT were also assessed, but none were noted in our study.

## Discussion

Patients undergoing amputation for PAD are usually at high risk for delayed wound healing and wound-related complications. The delay in wound healing increases the length of hospital stay and the financial burden. Our study included patients with atherosclerotic PAD undergoing lower extremity amputation. Most had high-risk factors for wound healing. With the use of iNPWT, there was a significant reduction in SSI (12% vs 36%; *p* = 0.047). Particularly in high-risk patients with uncontrolled DM, there was a decreased incidence of SSI in the iNPWT group compared with the standard dressing group.

Conventional dressings need to be changed twice a day or at least once daily, depending upon the soakage from the wound. During dressing changes, the sterile gauze may adhere to the wound, leading to denudation of the epithelium. By contrast, the iNPWT can be kept in place for 5–7 days. This significantly reduces the pain that a patient faces with daily dressing changes, and anticipated pain while changing the iNPWT can be managed pre-emptively.

In NPWT, the wound is covered using a polyurethane ether foam sponge secured in place using a semi-occlusive adhesive cover. This is then connected to a suction pump and fluid collection system. The suction pump applies between −50 and −175mmHg of either continuous or intermittent suction.^13,14^ Research on whether there is a benefit of using NPWT on closed wounds has shown controversial results.^15,16^ In a study by Kairinos *et al*, it was observed that increased interstitial pressure with application of NPWT leads to compression of the vessels, causing tissue hypoxia and increased velocity in the blood vessel.^17^ Tissue hypoxia causes vasodilatation and the release of vascular endothelial growth factors resulting in angiogenesis.^18^ Increased velocity in the blood vessel results in decreased intravascular hydrostatic pressure, which causes decreased efflux from the vessel resulting in reduced tissue oedema. The increased interstitial pressure also helps in splinting of the wound. iNPWT also decreases lateral tension on the wound by approximately 50%. Thus, iNPWT helps in wound healing by reducing lateral tension on the sutured wound, increasing blood flow, reducing tissue oedema and splinting.^18–20^

The use of iNPWT has been favoured across various surgical disciplines. In a prospective multicentre RCT by Stannard *et al*, which included 269 fractures, the risk of SSI in the control group was 1.9 times higher than in patients treated with iNPWT.^21^ In a retrospective study conducted by Mark *et al*, the use of iNPWT significantly reduced wound complications (0% vs 10.4%) in morbidly obese patients undergoing caesarean section.^22^ Galiano *et al* conducted a multicentre RCT in patients undergoing mammoplasty which showed a significant reduction in wound dehiscence (16.2% vs 26.4%).^23^

There is limited literature regarding the use of iNPWT in vascular surgery where patients are at high risk for wound-related complications. In a retrospective study by Zayan *et al*, the use of iNPWT decreased the incidence of SSI to 5.9% without any need for revision surgery.^24^ In the AIMS trial, the incidence of SSI following groin incision in vascular surgery typically performed for femoral artery exposure was studied. The results confirmed that use of iNPWT decreased the incidence of SSI significantly (13.2% vs 33.4%; *p* = 0.0015).^25^

Chang *et al* recently published a retrospective review of a prospectively maintained database of 54 patients undergoing major lower extremity amputation for PAD. They found that SSI incidence was significantly lower in the iNPWT group (13% vs 39%; *p* = 0.037).^26^ The results of our study are comparable with the abovementioned studies, with SSI being 12% in the iNPWT group and 36% in the standard dressing group. In a multicentre, prospective RCT, use of iNPWT on groin incisions after infra-inguinal bypass or femoral endarterectomy showed a similar incidence of SSI in an iNPWT group compared with a standard gauze group (11% vs 12%; *p* = 0.58).^27^ However, in a meta-analysis of RCTs, the use of iNPWT on groin incisions after vascular procedures reduced the rate of SSI, need for antibiotics and re-interventions.^28^ There was also a significant reduction in the duration for eligibility of prosthesis placement, which helps in the early rehabilitation of patients undergoing amputation. In addition, we noticed higher satisfaction in patients in the iNPWT group, which can be attributed to decreased discomfort or pain with fewer dressings needed. Based on our clinical experience with iNPWT after amputation in PAD patients, we have begun using it routinely at our institute.

### Study limitations

The sample size is small, which might increase the type II error of the study. This is a non-blinded study, which increases the chances of potential bias. There is a cost to using iNPWT, albeit much less than the costs involved with SSI and/or revision surgery. However, we were not able to perform an analysis of cost-effectiveness because our institute is state-funded.

## Conclusions

In high-risk patients with atherosclerotic PAD undergoing major limb amputations, the application of iNPWT helps in the reduction of wound-related complications and the time for rehabilitation.
